# Effect of vertical stopcock position on start-up fluid delivery in syringe pumps used for microinfusions

**DOI:** 10.1007/s10877-024-01156-z

**Published:** 2024-04-15

**Authors:** Markus Weiss, Pedro David Wendel-Garcia, Beate Grass, Maren Kleine-Brueggeney

**Affiliations:** 1 Department of Anesthesia, University Children’s Hospital, Steinwiesstrasse 75, 8032 Zurich, Switzerland; 2 Department of Intensive Care, University Hospital Zurich and University of Zurich, Zurich, Switzerland; 3 Newborn Research, Department of Neonatology, University Hospital Zurich and University of Zurich, Zurich, Switzerland; 4 Department of Cardiac Anesthesiology and Intensive Care Medicine, Deutsches Herzzentrum der Charité (DHZC), Berlin, Germany; 5 Charité - Universitätsmedizin Berlin, corporate member of Freie Universität Berlin and Humboldt-Universität Zu Berlin, Berlin, Germany

**Keywords:** Infusion, Microinfusion, Syringe, Pump, Start-up, Position

## Abstract

The purpose of this in vitro study was to evaluate the impact of the vertical level of the stopcock connecting the infusion line to the central venous catheter on start-up fluid delivery in microinfusions. Start-up fluid delivery was measured under standardized conditions with the syringe outlet and liquid flow sensors positioned at heart level (0 cm) and exposed to a simulated CVP of 10 mmHg at a set flow rate of 1 ml/h. Flow and intraluminal pressures were measured with the infusion line connected to the stopcock primarily placed at vertical levels of 0 cm, + 30 cm and − 30 cm or primarily placed at 0 cm and secondarily, after connecting the infusion line, displaced to + 30 cm and − 30 cm. Start-up fluid delivery 10 s after opening the stopcock placed at zero level and after opening the stopcock primarily connected at zero level and secondary displaced to vertical levels of + 30 cm and – 30 cm were similar (− 10.52 [− 13.85 to − 7.19] µL; − 8.84 [− 12.34 to − 5.33] µL and − 11.19 [− 13.71 to − 8.67] µL (*p* = 0.469)). Fluid delivered at 360 s related to 65% (zero level), 71% (+ 30 cm) and 67% (− 30 cm) of calculated infusion volume (*p* = 0.395). Start-up fluid delivery with the stopcock primarily placed at + 30 cm and − 30 cm resulted in large anterograde and retrograde fluid volumes of 34.39 [33.43 to 35.34] µL and − 24.90 [− 27.79 to − 22.01] µL at 10 s, respectively (*p* < 0.0001). Fluid delivered with the stopcock primarily placed at + 30 cm and − 30 cm resulted in 140% and 35% of calculated volume at 360 s, respectively (*p* < 0.0001). Syringe infusion pumps should ideally be connected to the stopcock positioned at heart level in order to minimize the amounts of anterograde and retrograde fluid volumes after opening of the stopcock.

## Introduction

Changeover of norepinephrine or epinephrine syringes in critically ill patients using microinfusions is associated with a high incidence of hemodynamic instability independent of the changeover strategy used [1, 2]. Underlying reasons for irregularities in fluid delivery during changeover include flow interruption caused by the changing maneuvre and delayed drug delivery when starting the new syringe infusion pump at low flow rates [3–6].

Recent publications revealed that the level of central venous pressure (CVP) as well as the vertical position of the pump connected to the central venous catheter can cause anterograde or retrograde fluid shifts when opening the stopcock resulting in a bolus or in delayed drug delivery [7–11]. The course and amount of fluid shift is related to the direction and degree of pressure gradient between the central venous catheter and the newly connected infusion pump assembly [10].

The vertical position of the stopcock connecting the line of the syringe infusion pump primed for example with a catecholamine to the central venous catheter is not standardized and the stopcock may be placed at various levels, ranging from the level of the legs (particularly with femoral central venous access) to the upper boarder of the bed (particularly with jugular central venous access and head-elevated patient positioning). The impact on start-up fluid delivery of microinfusions of these varying vertical positions and of the displacement of the stopcock when connecting the syringe infusion pump assembly has not been evaluated. Thus, the purpose of this study was to evaluate start-up fluid delivery after connecting the syringe infusion pump assembly before or after placing the stopcock at different vertical levels.

## Methods

### Study setting and experiments performed

Start-up fluid delivery and intraluminal pressures were simultaneously assessed by means of liquid flow sensors and invasive pressure transducers in an in vitro study design.

To this end, infusion syringes of 50 mL volume (BD Plastipak 50 mL, Becton Dickinson and Company, Drogheda, Ireland) with an infusion line extension (Extension Set with Pressure Disk, PE/PVC, set length 200 cm, bore 0.9 mm, priming volume 1.5 mL, CareFusion, Rolle, Switzerland) were filled with sterile distilled water (1000 mL Plastipur, Fresenius Kabi AG, Bad Homburg, Germany). To avoid deposits within the liquid flow sensor, impairing fluid flow measurements, distilled water rather than physiological saline solution was used, as recommended by the manufacturer. The syringes were placed in a conventional syringe pump (BD Alaris™ CC Plus Guardrails Syringe Pump, Rolle, Switzerland) and the pressure disk inserted in the corresponding disk holder.^10^ The extension line was connected to a two-way stopcock (Discofix, B. Braun, Melsungen AG, Melsungen, Germany) linked to the inlet of the flow sensor by means of a 50 cm stiff infusion line (Original Perfusor Line, B. Braun, Melsungen, Germany). The flow sensor outlet was connected directly to the medial lumen (22 Ga.) of a 8 cm long, 5.5 Fr. sized pediatric three-lumen central venous catheter (Pediatric Three-Lumen CVC, Arrow, Teleflex Medical, Athlone, Ireland) placed in a fluid chamber. The fluid chamber was filled with distilled water and placed with the fluid surface level 13 cm above the in-/outlet level of the flow sensor to simulate a central venous pressure (CVP) of 10 mmHg.

Start-up fluid delivery was measured with the syringe outlet placed at the in-/outlet level of the liquid flow sensors (SLI-1000; Sensirion AG, Staefa, Switzerland) which were placed at heart level (zero level) and exposed to a simulated CVP of 10 mmHg. Fluid flow values measured at intervals of 100 ms and continuously averaged for 50 values (5 s) as well as the total amount of fluid delivered were simultaneously displayed on a personal computer using a specific software provided by the manufacturer (Sensirion_Software_USB_RS485_Sensor_Viewer_V2.91). The software`s totalizer functionality allowed to continuously summarize and display amounts of fluid delivered through the sensor. Displayed data were continuously recorded and stored for later analysis using the Movavi Screen Recorder Software 2022.

**Fig. 1:**
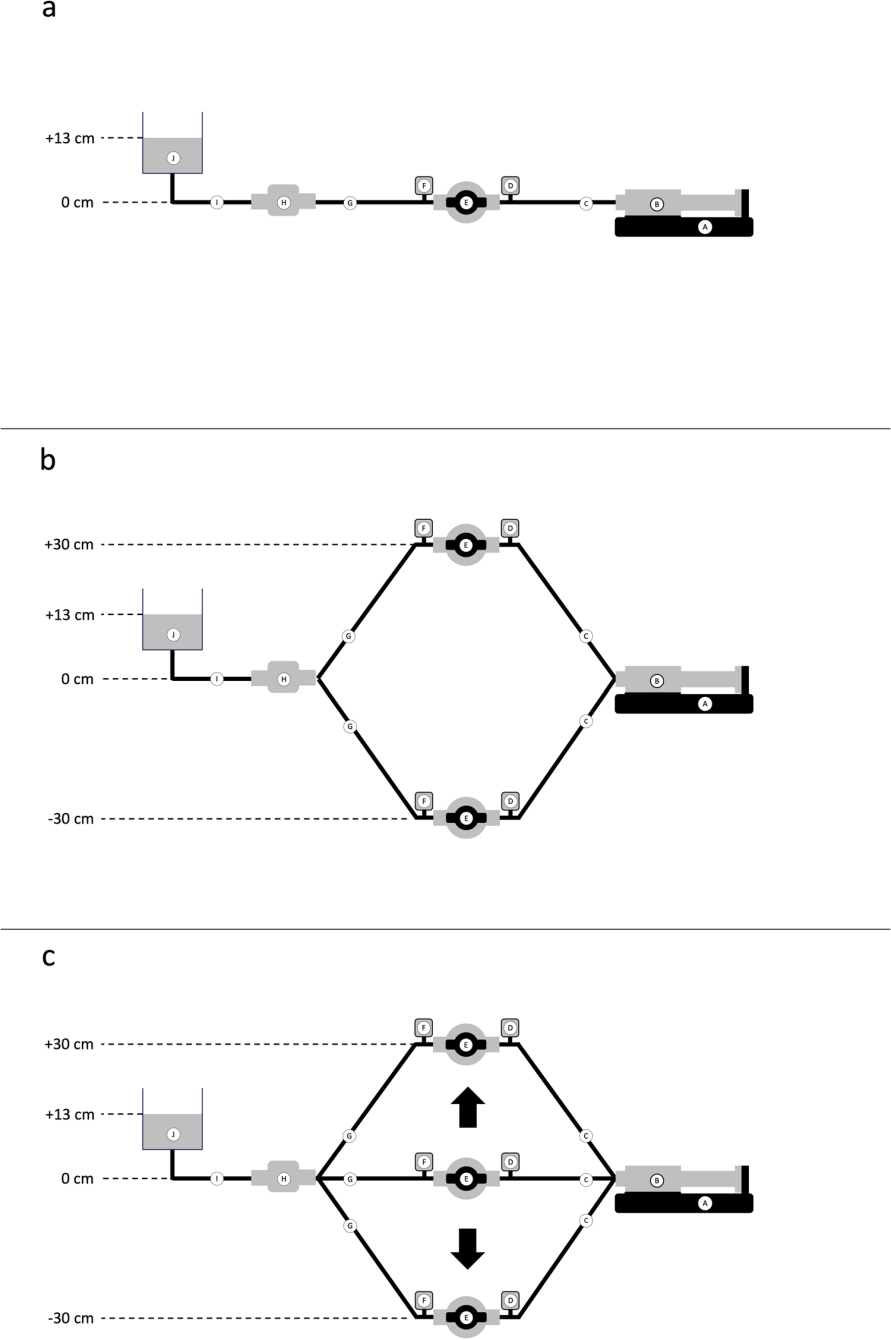
**a-c** Schematic of the experimental in vitro set-up for simultaneous assessment of start-up fluid delivery and intraluminal pressures of a conventional syringe infusion pump assembly with the connecting stopcock placed at or displaced to vertical positions levels of 0 cm, + 30 cm and – 30 cm. **A** Syringe pump; **B** 50 mL infusion syringe; **C** 200 cm afferent extension line; **D** pre-stopcock (afferent) pressure transducer; **E** two-way stopcock; **F** post-stopcock (efferent) pressure transducer; **G** 50 cm efferent extension line; **H** liquid flow sensor; **I** 22 Ga. medial limb of a pediatric 5.5 Fr. 8 cm three-lumen central venous catheter; **J** fluid chamber filled with distilled water and placed with the fluid surface 13 cm above level of syringe outlet and sensor in-/outlet. **a** Control set-up (the afferent infusion line is connected to the stopcock placed at zero (heart) level. **b** Set-up 1 (the afferent infusion line is connected to the stopcock primarily placed at vertical levels of + 30 cm or – 30 cm). **c** Set-up 2 (the afferent infusion line is primarily connected to the stopcock placed first at zero (heart) level and then the stopcock is secondarily displaced to vertical levels of + 30 cm or – 30 cm)

Pressure transducers (TruWave; Edwards Life-Sciences, Irvine, CA, USA) were included to allow for measurements of intraluminal pressures in front of (afferent limb) and behind (efferent limb) the two-way stopcock connecting the infusion line of the syringe pump and the central venous catheter. The pressure transducers were connected to a vital sign monitor (S/5TM Anesthesia Monitor; Datex-Ohmeda, Helsinki, Finland) to simultaneously display intraluminal pressures during the different manipulations described in the experiments below. The experimental set-up is visualized in Fig. 1.

#### Control set-up: placement of stopcock at vertical level of 0 cm (Fig. 1a)

This set-up mimicks the clinical scenario with the patient`s upper body in a head-up position when the afferent infusion line is connected to a stopcock positioned at heart level. The primed afferent infusion line was purged using a 1 ml fluid bolus by means of the pump’s bolus function with the distal end of the afferent infusion line held at zero level to eliminate mechanical gaps [5]. One minute after purging, the afferent infusion line was connected to the inlet of the stopcock placed at zero level. The assessment of fluid flow was initiated, the two-way stopcock opened and the pump activated at a flow rate of 1 ml/h.

#### Set-up 1: primary placement of stopcock at vertical levels of + 30 cm or – 30 cm (Fig. 1b)

This set-up mimicks the clinical scenario with the patient`s upper body in a head-up position when the afferent infusion line is connected to the stopcock placed at the upper border of the mattress (for example in case of jugular central venous access, set-up with stopcock at + 30 cm) and placed at the horizontal part of the mattress next to the patients leg (for example in case of femoral central venous access, set-up with stopcock at − 30 cm).

The primed afferent infusion line was purged using a 1 ml fluid bolus by means of the pump’s bolus function to eliminate mechanical gaps. During this purging, the distal end of the afferent infusion line was held at the inlet level of the stopcock, placed at vertical levels of + 30 cm or –30 cm from heart level. One minute after purging, the afferent infusion line was connected to the inlet of the stopcock-pressure transducer assembly. Afterwards, the assessment of fluid flow was initiated, the two-way stopcock opened and the pump activated at a flow rate of 1 ml/h.

#### Set-up 2: secondary placement of stopcock from zero vertical level to + 30 cm or – 30 cm (Fig. 1c)

This set-up mimicks the clinical scenario with the patient`s upper body in a head-up position when the afferent infusion line is connected to the stopcock held at heart level and secondarily placed at the upper border of the mattress (for example in case of jugular central venous access, + 30 cm) and placed at the horizontal part of the mattress next to the patients leg (for example in case of femoral central venous access, − 30 cm), respectively. The primed afferent infusion line was purged using 1 ml fluid bolus by means of the pump’s bolus function to eliminate mechanical gaps with the distal end of the afferent infusion line held at zero level. One minute after purging, the afferent infusion line was connected to the inlet of the stopcock placed at zero level. Then, the stopcock-pressure transducer assembly was displaced to vertical levels of + 30 cm or –30 cm, the assessment of fluid flow initiated, the two-way stopcock opened and the pump activated at a flow rate of 1 ml/h.

### Measurements

Experiments were repeated five times with two identical syringe infusion pumps (A and B) for each set-up and vertical position in randomized order (www.random.org). Experiments were performed at room temperature between 22.0 and 23.0 °C. The Faststart option and the to-keep-venous-open functionality (TKVO) were inactivated for the assessments.

### Outcome measures

Primary outcome parameters included antero- and retrograde infusion volumes measured at 10, 60, 180 and 360 s after opening the two-way stopcock and activating the pump`s start button.

Secondary outcome parameters were simultaneously measured intraluminal pressures in front of (P_1_; pre-stopcock; afferent limb) and behind (P_2_; post-stopcock; efferent limb) the two-way stopcock assessed one minute after the following procedures: initial positioning of the stopcock, before connection of the afferent infusion line to the stopcock (T_1_), after connection of the afferent infusion line to the stopcock (T_2_), after vertical displacement of the stopcock in set-up 2 (T_3_), and after opening of the two-way stopcock (T_4_).

### Statistical analysis

Longitudinal analysis of delivered fluid over time was approached by means of hierarchical linear mixed effects model analysis. As independent variable fixed effects time point, modelled by means of a natural cubic spline with two degrees of freedom, set-up and position were entered into the model, with a three-way interaction term. As random effects, intercepts for subjects nested in pumps were employed. P values were calculated using Satterthwaite’s method. The intraclass correlation coefficient (ICC) was calculated to determine the data variance attributable to the pump specimen (A/B). Statistical analysis was performed through a fully scripted data management pathway using the R environment for statistical computing version 4.3.2 (R Core Team, Vienna, Austria). Data are presented as means with 95% confidence intervals.

## Results

### Primary outcome parameters

**Fig. 2:**
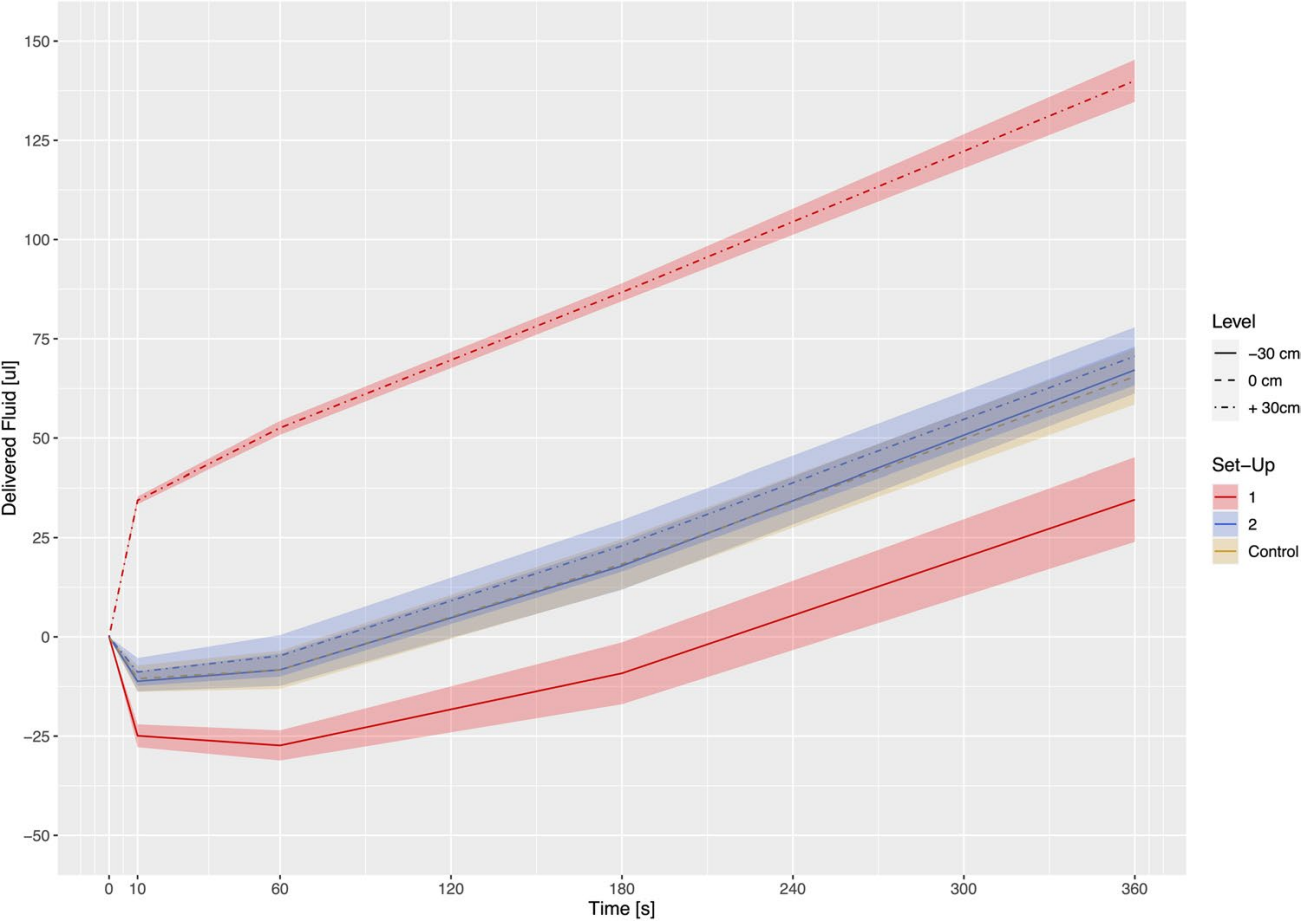
Graphical illustration of the time-varying effect on fluid delivered after opening the two-way stopcock and starting the syringe infusion pump at an infusion rate of 1 mL/h with the afferent infusion line connected to the stopcock placed at zero level (control set-up), connected to the stopcock primarily placed at vertical levels of − 30 cm and + 30 cm (set-up 1) as well as connected to the stopcock placed first at zero level and secondarily displaced to vertical levels of -30 cm and + 30 cm (set-up 2). Estimated effects are presented as lines, 95% confidence intervals are presented as shaded colored areas

Start-up fluid delivery at 10 s and 360 s after opening the stopcock were similar in the set-up with the stopcock placed at zero level (control set-up) and in the set-up with the stopcocks primarily connected at zero level and secondarily displaced to vertical levels of + 30 cm and − 30 cm (set-up 2) (− 10.52 [− 13.85 to − 7.19] µL; − 8.84 [− 12.34 to − 5.33] µL and − 11.19 [− 13.71 to − 8.67] µL (*p* = 0.469) and 65.49 [58.49 to 72.49] µL; 70.60 [63.28 to 77.92] µL and 67.14 [61.23 to 73.05] µL (*p* = 0.395)) (Table 1; Fig. 2). The delivered fluid at 360 s related to 65% (zero level), 71% (+ 30 cm) and 67% (− 30 cm) of calculated infusion volume delivered at 1 mL/h set flow rate.

**Table 1:** Infusion volumes delivered 10, 60, 180 and 360 s after opening the two-way stopcock and activation of the pumps`s start button at an infusion flow rate of 1 ml/h with connection of the afferent infusion line to the stopcock placed at zero level (control set-up), to the stopcock primarily placed at vertical levels of + 30 cm and − 30 cm (set-up 1) and to the stopcock first placed at zero level and secondarily displaced to vertical levels of + 30 cm and − 30 cm (set-up 2)

Experimental set-up	Stopcock position	Infusion volume delivered (µL) Percentage of delivered versus calculated infusion volume (%)			
	cm	at 10 s	at 60 s	at 180 s	at 360 s
Control	0	− 10.52 [− 13.85 to − 7.19] − 379%	− 8.31 [− 13.10 to − 3.52] − 50%	18.35 [12.05 to 24.66] 37%	65.49 [58.49 to 72.49] 65%
1	+ 30	34.39 [33.43 to 35.34] 1238%	52.55 [50.78 to 54.32] 315%	86.72 [84.46 to 88.98] 173%	140.02 [134.71 to 145.32] 140%
1	− 30	− 24.90 [− 27.79 to − 22.01] − 896%	− 27.35 [− 31.15 to − 23.55] − 164%	− 9.17 [− 16.96 to − 1.38] − 18%	34.56 [23.95 to 45.18] 35%
2	+ 30	− 8.84 [− 12.34 to − 5.33] − 318%	− 4.77 [− 9.99 to 0.46] − 29%	22.93 [16.45 to 29.40] 46%	70.60 [63.28 to 77.92] 71%
2	− 30	− 11.19 [− 13.71 to − 8.67] − 403%	− 8.30 [− 12.36 to − 4.23] − 50%	17.88 [11.87 to 23.90] 36%	67.14 [61.23 to 73.05] 67%

Data presented as mean [CI 95%] and percentages of measured versus calculated infusion volumes

Start-up fluid delivery at 10 s after opening the stopcock in the set-up with the stopcock primarily placed at + 30 cm and − 30 cm (set-up 1) resulted in large anterograde and retrograde fluid volumes of 34.39 [33.43 to 35.34] µL and − 24.90 [− 27.79 to − 22.01] µL, respectively (*p* < 0.0001). In this set-up, fluid delivered after 360 s was 140% (stopcock at + 30 cm) and 35% (stopcock at − 30 cm) of calculated volume (*p* < 0.0001).

### Secondary outcome parameters

The course of intraluminal post-stopcock pressures (P_2_; efferent limb) in all set-ups was related to CVP and vertical level of the stopcock (Table 2). Regarding the pre-stopcock intraluminal pressures (P_1_; afferent limb), connection of the afferent infusion line to the stopcock resulted in an increase from zero to mean pressures of 5.0, 5.5 and 5.3 mmHg in the set-up with stopcocks primarily positioned at zero level (control set-up and set-up 2). In contrast, pre-stopock intraluminal pressures (P_1_) in set-up 1 with the stopcock positioned at + 30 cm and − 30 cm) increased from zero to mean pressures of 27.0 and 24.6 mmHg, respectively, when the afferent infusion line was connected. Accordingly, pressure gradients were similar at stopcocks positioned at zero level (− 5.0 [− 5.51 to − 4.49] mmHg, − 4.5 [− 5.17 to − 3.83] mmHg and − 4.7 [− 5.21 to − 4.19] mmHg). In contrast, pressure gradients after connecting the afferent infusion line to the stopcock positioned at vertical levels of + 30 cm and − 30 cm were 39.0 [37.32 to 40.68] mmHg and − 7.4 [8.07 to − 6.73] mmHg, respectively. Pressure gradients during displacement of stopcocks to vertical levels of + 30 cm and − 30 cm (set-up 2) slightly increased and decreased by about 10%, respectively.

**Table 2:** Intraluminal pressures simultaneously measured in front of (pre-stopcock; afferent limb; P_1_) and behind (post-stopcock; efferent limb; P_2_) the two-way stopcock placed at zero level (control set-up), the two-way stopcock primarily placed at vertical levels of + 30 cm and − 30 cm (set-up 1) as well as the two-way stopcock placed first at zero level and secondarily displaced to vertical levels of + 30 cm and − 30 cm (set-up 2)

Experimental Set-up	Primary stopcock position	Stopcock pressures before connecting (T_1_)		Stopcock pressures after connecting (T_2_)		Pressure gradients (T_2_)	Secondary stopcock position	Stopcock pressures after displacement (T_3_)		Pressure gradients before opening	Pressures after opening (T_4_)	
		P_1_	P_2_	P_1_	P_2_	P_1_-P_2_		P_1_	P_2_	P_1_-P_2_	P_1_	P_2_
	cm	mmHg	mmHg	mmHg	mmHg	mmHg	cm	mmHg	mmHg	mmHg	mmHg	mmHg
Control	0	0.1 [− 0.09 to 0.29]	10.0 [10.0 to 10.0]	5.0 [4.49 to 5.51]	10.0 [10.0 to 10.0]	− 5.0 [− 5.51 to − 4.49]	0	n/a	n/a	− 5.0 [− 5.51 to − 4.49]	9.7 [9.40 to 9.99]	10.0 [10 to 10]
1	+ 30	0.0 [0 to 0]	− 12.0 [− 12.0 to − 12.0]	27.0 [25.32 to 28.68]	− 12.0 [− 12.0 to − 12.0]	39.0 [37.32 to 40.68]	+ 30	n/a	n/a	39.0 [37.32 to 40.68]	− 12.1 [− 12.29 to − 11.09]	− 12.0 [− 12.0 to − 12.0]
	− 30	0.0 [0 to 0]	31.9 [31.70 to 32.1]	24.6 [23.93 to 25.27]	32.0 [32.0 to 32.0]	− 7.4 [− 8.07 to − 6.73]	− 30	n/a	n/a	− 7.4 [− 8.07 to − 6.73]	31.70 [31.40 to 32.0]	32.0 [32.0 to 32.0]
2	0	0.0 [0 to 0]	10.0 [10.0 to 10.0]	5.50 [4.83 to 6.17]	10.0 [10.0 to 10.0]	− 4.5 [− 5.17 to − 3.83]	+ 30	− 16.0 [− 16.92 to − 15.08]	− 12.0 [− 12.0 to − 12.0]	− 4.0 [− 4.92 to − 3.08]	− 12.1 [− 12.29 to − 11.9]	− 12.0 [− 12.0 to − 12.0]
	0	0.0 [0 to 0.3]	10.0 [10.0 to 10.0]	5.30 [4.79 to 5.81]	10.0 [10.0 to 10.0]	− 4.7 [− 5.21 to − 4.19]	− 30	26.8 [26.16 to 27.44]	32.0 [32.0 to 32.0]	− 5.2 [− 5.84 to − 4.56]	31.8 [31.54 to 32.06]	32.0 [32.0 to 32.0]

Pressure values were noted before connecting (T_1_) and after connecting (T_2_) the afferent infusion line to the stopcock (T_2_), after vertical displacement of the stopcock in set-up 2 (T_3_), and after opening the two-way stopcock (T_4_). Pressure gradients (P_1_-P_2_) were calculated after connecting (T_2_) and before opening the two-way stopcock. Data presented as mean [CI 95%]

*n/a* not applicable

### Correlation of 10-s fluid volumes with pressure gradients

**Fig. 3:**
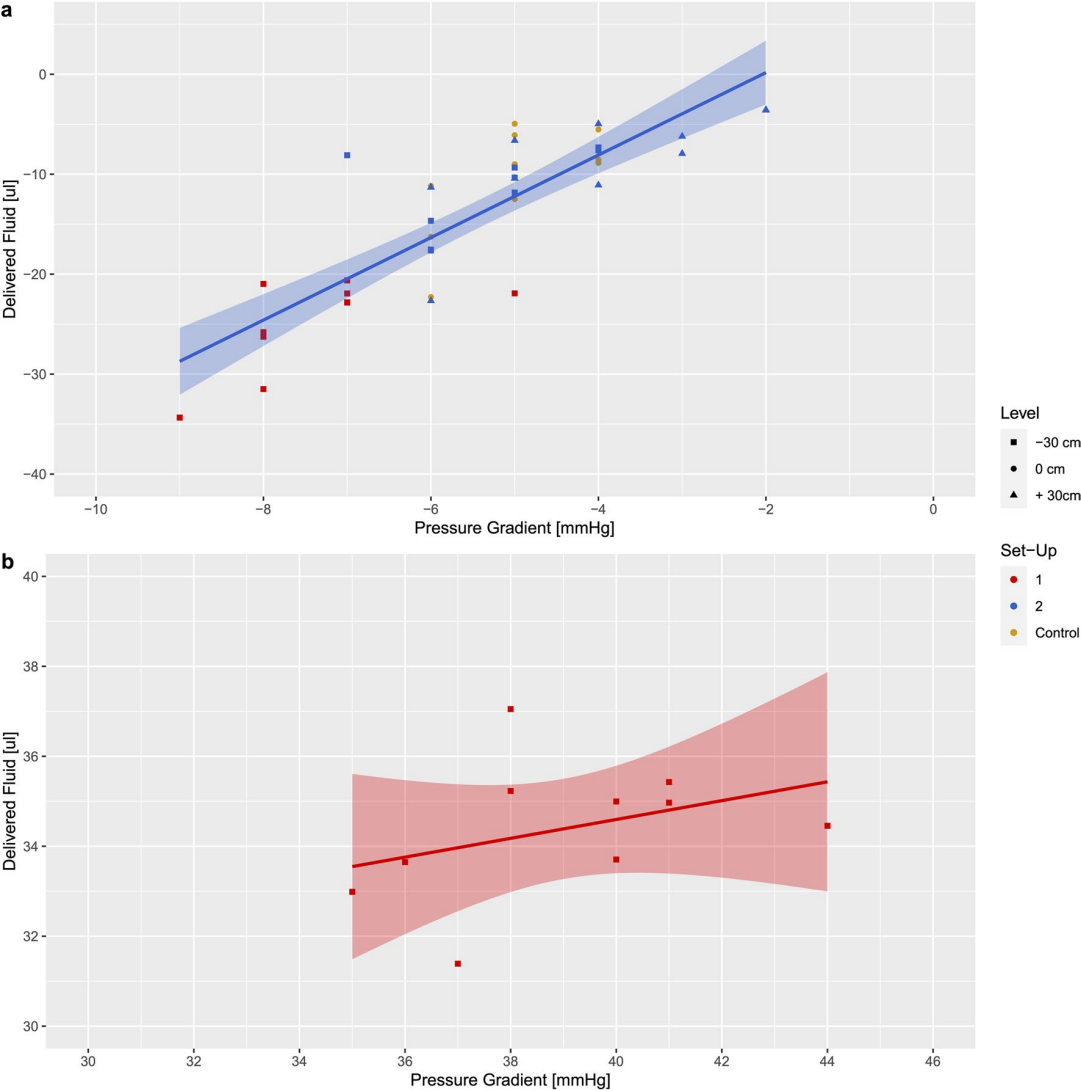
Linear regression plots for the correlation between 10-s fluid volumes delivered after opening the stopcock versus pressure gradients measured between “afferent infusion line and efferent central venous catheter line” before opening the stopcock placed at zero level (control set-up), the stopcock primarily placed at vertical levels of + 30 cm and − 30 cm (set-up 1) as well as the stopcock placed first at zero level for afferent infusion line connection and secondarily displaced to vertical levels of + 30 cm and − 30 cm (set-up 2). **a** 10 s retrograde fluid volumes (R^2^ = 0.713, *n* = 40); **b** anterograde fluid volumes (R^2^ = 0.026; *n* = 10)

Retrograde fluid volumes delivered in the first 10 s after opening the stopcock correlated well with pressure gradients before opening the stopcock (r^2^ = 0.713; Fig. 3a), while 10-seconds anterograde volumes did not (r^2^ = 0.026); Fig. 3b). The intraclass correlation coefficient (ICC) between pump A and B was 0.037, indicating a very low effect on measured differences attributable to the pump A or B.

## Discussion

This in vitro assessment evaluated the effect of the level of the stopcock connecting afferent infusion line of the syringe infusion pump and efferent central venous line as well as the effect of the timing of displacement of the stopcock on start-up fluid delivery of microinfusions. The main findings are that start-up fluid delivery is similar when the stopcock is positioned at zero level and when the stopcock is connected first at zero level and then displaced to a higher or lower level. This was in contrast to connecting the afferent infusion line to the stopcock primarily placed to a higher or lower vertical level, which resulted in considerable antero- and retrograde fluid volumes as well as increased or reduced total fluid delivery 360 s after opening the stopcock and starting the pump.

The measured start-up fluid delivery with retrograde fluid volumes after opening the stopcock in the control set-up as well as the measured pressure increase after connecting the afferent infusion line to the stopcock are in agreement with a prior report with the stopcock and syringe infusion pump assembly placed at zero level and exposed to a CVP of 10 mmHg [10]. The increase of intraluminal pressure is thought to be caused by displacement of fluid into the syringe infusion pump assembly when the distal end of the afferent infusion line is screwed into the infusion port of the closed stopcock. Thereby, the pressure increase is mainly dependent on the fluid volume displaced and the compliance of the syringe infusion pump assembly. Basically, this pressure increase when connecting the afferent infusion line to the infusion port of a stopcock counteracts the efferent infusion line pressure, mainly determined by the level of CVP; otherwise even higher pressure gradients from the central venous system to the syringe infusion pump assembly with higher backflow volumes and more prolonged lag-times in drug delivery from the syringe infusion pump would result. [11].

Based on the findings of this in vitro study, pre-to-post-stopcock pressure gradients after connecting the afferent infusion line remained nearly constant during lowering and elevating the stopcock resulting in similar start-up fluid delivery compared to the control set-up inspite of large differences in absolute intraluminal pre- and post-stopcock pressures. In contrast, intraluminal pressure changes in the infusion port of the stopcock primarily placed at vertical levels of + 30 cm and − 30 cm were five times higher than at zero level when connecting the afferent infusion line. This finding confirms earlier observations that priming the extension line with its distal end held above or below the vertical level of the syringe outlet leads to a higher pressure increase when connecting the line to the stopcock when compared to zero level conditions [10]. The observation may be explained that the hydrostatic pressure between infusion syringe outlet and primed infusion line end affects volume-pressure characteristics of the syringe pump assembly by traction and compression respectively. Consecutively in the current study, this resulted in pressure gradients with the stopcock primarily placed at a vertical level of + 30 cm being 39 cmH_2_0 and with the stopcock placed at a level of − 30 cm being higher (− 7.4 cmH_2_O) than expected.

Retrograde fluid volumes correlated well with intraluminal pressure gradients ranging from − 2 cmH_2_0 to − 9 cmH_2_0. This was in contrast to anterograde fluid volumes that did not correlate to the much higher pressure gradients (33 cmH_2_O to 44 cmH_2_O). It is possible that higher pressure gradients associated with higher fluid velocities lead to turbulent flow conditions with an increased resistance, while with lower pressure gradients a laminar flow was present. This is supported by the fact that anterograde fluid volumes were lower than could be expected in view of the high pressure gradients.

It could be suggested that asymmetrical afferent and efferent limbs of the tubing attached to the stopcock may be the reason for different retrograde/anterograde flows as the stopcock level changes. This could particularly be assumed for a setting similar to experimental set-up 2, when the stopcock connected to asymmetrical afferent and efferent limbs made from very compliant tubing is elevated or lowered [12]. Subsequent fluid would be siphoned in or emptied from the smaller to the larger compartment.

However, in this study only stiff infusion line extensions were used, and as shown in experimental set-up 2 lowering or elevating the stopcock had no significant effect on fluids delivered (Table 1) as well as no significant effect on pressure gradients (Table 2) when compared to the control set-up. As demonstrated by a recent study, also using asymmetrical afferent and efferent stiff tubing attached to the stopcock, no acute fluid shifts were measured after opening the stopcock when intraluminal pressures between the afferent to efferent limb were equilibrated, even with the syringe pump placed below or above the level of the stopcock [13]. All these data suggest that pressure gradients between afferent and efferent limb connected to the stopcock are responsilble for the antero- and retrograde fluid delivery measured.

The findings of this study have important clinical implications. In critical care medicine and anesthesia, when vasoactive and/or inotropic drugs are administered by means of microinfusions the vertical level of stopcock placement is not standardized. Even more, displacement of a stopcock or a multifold-stopcock assembly is not regarded to be a critical maneuver that could cause fluid flow irregularities as long as stiff infusion lines and incompliant infusion ports are used [12]. This is in contrast to the whole syringe infusion pump assembly, which should not be vertically displaced relative to the patient level in order to avoid fluid flow irregularities [5, 14, 15]. So far, CVP and vertical pump level have been shown to affect direction and extent of start-up fluid delivery after opening the stopcock [7, 9, 10]. Based on the current study results vertical stopcock position may also significantly affect start-up fluid delivery. The results are of particular relevance during changeover of syringe infusion pumps used for the administration of highly concentrated vasoactive and inotropic drugs with short half-life times at low flow rates. Overinfusion of 300% in the first 60 s and zero drug delivery within the first 180 s with only 30% of calculated drug amount delivered after 360 s are likely to cause acute episodes of hypertension or hypotension, respectively [1, 2]. Therefore, syringe infusion pumps used for microinfusion should be connected to a stopcock placed at patient heart level in order to minimize anterograde and retrograde fluid volumes after opening the stopcock and starting the pump. Subsequent displacement of the stopcock seems not to represent a relevant problem.

This in vitro study only used one brand of a syringe infusion pump assembly. The compliance of infusion syringes and pump drivers, the resistance of extension lines, central venous catheters and stopcocks, and the viscosity of the infusate may vary with size and brand [5, 14–17]. Furthermore, dead space volumes of catheters, extension lines, stopcocks and filters cause further lag times in drug delivery during pump start-up, which are additionally affected whether carrier infusion are used or not [6, 18, 19]. All these factors may, together with various vertical levels of the stopcock, affect start-up fluid delivery. Nevertheless, the current study using standard infusion equipment clearly demonstrated a significant impact of the vertical stopcock position on start-up fluid delivery in microinfusions.

## Conclusions

When connecting and starting syringe infusion pumps with microinfusions, the vertical level of the stopcock has considerable effects on fluid delivery. Ideally, syringe infusion pumps should be connected to the stopcock positioned at heart level in order to minimize anterograde and retrograde fluid volumes after opening the stopcock and starting the pump. Secondary displacement of the stopcock after connecting the infusion line is not a relevant issue.

## Data Availability

The data that support the findings of this study are available from the corresponding author upon reasonable request.
